# The epidemiology and clinical features of melioidosis in Far North Queensland: Implications for patient management

**DOI:** 10.1371/journal.pntd.0005411

**Published:** 2017-03-06

**Authors:** James D. Stewart, Simon Smith, Enzo Binotto, William J. McBride, Bart J. Currie, Josh Hanson

**Affiliations:** 1 Department of Medicine, Cairns Hospital, Cairns, Queensland, Australia; 2 James Cook University Clinical School, Cairns Hospital, Cairns, Queensland, Australia; 3 Infectious Diseases Department, Royal Darwin Hospital, Darwin, Northern Territory, Australia; 4 Global and Tropical Health Division, Menzies School of Health Research, Charles Darwin University, Darwin, Northern Territory, Australia; 5 The Kirby Institute, University of New South Wales, Sydney, New South Wales, Australia; University of California San Diego School of Medicine, UNITED STATES

## Abstract

**Background:**

The epidemiology, clinical presentation and management of melioidosis vary around the world. It is essential to define the disease’s local features to optimise its management.

**Principal findings:**

Between 1998 and 2016 there were 197 cases of culture confirmed melioidosis in Far North Queensland; 154 (78%) presented in the December-April wet season. 145 (74%) patients were bacteraemic, 58 (29%) were admitted to the Intensive Care Unit and 27 (14%) died; nine (33%) of these deaths occurred within 48 hours of presentation. Pneumonia was the most frequent clinical finding, present in 101 (61%) of the 166 with available imaging. A recognised risk factor for melioidosis (diabetes, hazardous alcohol use, chronic renal disease, chronic lung disease, immunosuppression or malignancy) was present in 148 (91%) of 162 patients with complete comorbidity data. Despite representing only 9% of the region’s population, Aboriginal and Torres Strait Island (ATSI) people comprised 59% of the cases. ATSI patients were younger than non-ATSI patients (median (interquartile range): 46 (38–56) years versus 59 (43–69) years (p<0.001) and had a higher case-fatality rate (22/117 (19%) versus 5/80 (6.3%) (p = 0.01)). In the 155 patients surviving the initial intensive intravenous phase of treatment, eleven (7.1%) had disease recurrence, despite the fact that nine (82%) of these patients had received prolonged intravenous therapy. Recurrence was usually due to inadequate source control or poor adherence to oral eradication therapy. The case fatality rate declined from 12/44 (27%) in the first five years of the study to 7/76 (9%) in the last five (p = 0.009), reflecting national improvements in sepsis management.

**Conclusions:**

Melioidosis in Far North Queensland is a seasonal, opportunistic infection of patients with specific comorbidities. The ATSI population bear the greatest burden of disease. Although the case-fatality rate is declining, deaths frequently occur early after hospitalisation, reinforcing the importance of prompt, targeted therapy in high-risk patients.

## Author summary

*Burkholderia pseudomallei* is endemic to the tropics and is responsible for the disease melioidosis. Exposure rarely evolves to significant disease in the absence of specific comorbidities. Conversely, in susceptible hosts, the disease can be rapidly fatal if unrecognised. Patients require an extended course of intravenous and oral antibiotic therapy to treat the disease and to prevent its recurrence. This retrospective, case series describes the epidemiology, presentation and outcomes of 197 cases of melioidosis in Far North Queensland (FNQ), Australia. The study confirms that melioidosis is predominantly a seasonal, opportunistic infection occurring among patients with specific comorbidities that include diabetes mellitus, hazardous alcohol use, chronic kidney disease, chronic lung disease, malignancy and immunosuppressive therapy. Melioidosis is more common in the local Aboriginal and Torres Strait Islander (ATSI) population, which has a higher prevalence of these comorbidities. Although overall case-fatality rates are declining, they are higher in the ATSI population, reflecting ongoing socioeconomic disadvantage. Disease recurrence is more likely in patients with inadequate source control and suboptimal adherence, factors that are not necessarily mitigated by extending the duration of intravenous therapy.

## Introduction

*Burkholderia pseudomallei*, an environmental bacterium endemic to the tropics, is responsible for the disease melioidosis [1]. Serological studies suggest that *B*. *pseudomallei* infection is usually asymptomatic; however, it may also cause a rapidly fatal illness, particularly in those with co-morbidities [2, 3]. Pneumonia is the commonest presentation, however almost any organ can be involved with the liver, spleen, kidney and prostate regularly affected [1, 4]. Patients with melioidosis are frequently bacteraemic and often present in septic shock, but solitary skin lesions without systemic manifestations are a common presentation in children and adults without risk factors [1].

The epidemiology and clinical manifestations of the disease vary in different geographical locations [1, 5]. This may be explained by differences in the local prevalence of comorbidities and variable access to sophisticated diagnostic facilities. The route of infection (percutaneous, inhalation or ingestion) is increasingly thought to also have a major influence on presentation [6]. These geographical differences in the clinical features of melioidosis have implications for empirical management of sepsis. There are also ramifications for the disease’s relatively complex treatment regimen: a minimum of 10–14 days of intravenous antibiotics (intensive phase) followed by at least 3 months of oral antibiotics (eradication phase) is usually recommended, but in cases of extensive or complicated disease, an even longer duration of antibiotic therapy is advised [7].

However, even with adherence to these treatment recommendations, disease relapse is still seen. In one series from the Northern Territory of Australia, 4.3% of patients surviving the intensive phase of therapy had molecularly confirmed relapse [8]. In a Thai series, the relapse rate was 9.3% [9]. This relatively high rate of relapse—and the recognition that there is frequently poor adherence to the long course of oral therapy—has led to a suggestion that patients may benefit from an extended phase of intravenous therapy. A longer phase of intravenous therapy was associated with very low relapse rates in a recent series from Darwin [10]. However, while there are plausible arguments for this strategy, it has not been evaluated in other locations.

This retrospective case series was performed to define the epidemiology, presentation, clinical course and response to treatment of melioidosis in Far North Queensland in Australia. In addition, by correlating local relapse rates with the duration of intensive intravenous therapy, it aimed to determine if the apparent efficacy of the Darwin treatment guideline could be demonstrated at an independent site.

## Methods

**Fig 1:**
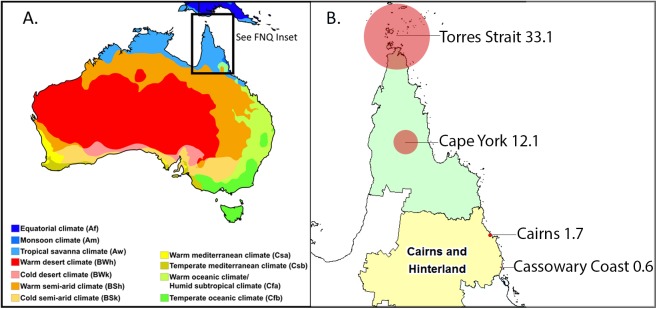
A. Australia climate map [12]. B. Far North Queensland: annual disease incidence by region (expressed per 100,000 population) and Queensland Health Service Districts [41].

Cairns Hospital is a 531 bed, tertiary referral hospital located in the tropical Far North of the state of Queensland, Australia. It serves a population of approximately 280,000 people, 9% of whom identify as Aboriginal or Torres Strait Islanders (ATSI) (Fig 1) [11]. Patients were eligible for inclusion in the study if they had a positive laboratory culture for *B*. *pseudomallei* in Cairns Hospital laboratory, the sole microbiology provider for FNQ health services, between January 1, 1998 and July 31, 2016

**Fig 2:**
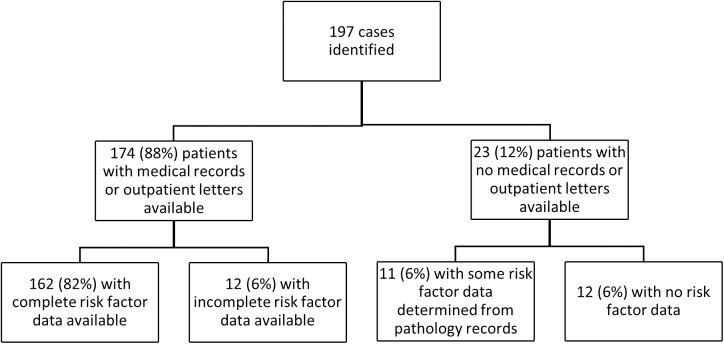
Data collection and its completeness.

The hospital medical record of each case was reviewed to determine the patients’ demographics, their clinical presentation, their comorbidities and their disease course (Fig 2). Hazardous alcohol use was said to be present if it had been documented in the medical record in the 12 months prior to presentation. Chronic lung disease was said to be present if a patient was receiving any ongoing treatment for a chronic lung condition. Chronic kidney disease was said to be present if there had been a serum creatinine ≥ 150 μmol/L documented before the presentation. Immunosuppression was said to be present if the patent was using immunosuppressive agents, including corticosteroids, chemotherapy, or immunomodulatory therapies. The presence of an active malignancy was also recorded. Admission to the Intensive Care Unit (ICU), the use of mechanical ventilation and renal replacement therapy (RRT) was documented; septic shock was defined as a requirement for vasopressors. The duration of the intensive and proposed eradication phase of treatment was also documented; this was compared to published recommendations from Darwin [10]. Adherence to eradication therapy was determined from the medical record and outpatient letters. The state-wide laboratory database was examined to identify culture confirmed treatment failure.

Death attributable to melioidosis within 90 days of diagnosis was documented. Disease recurrence was defined as clinical deterioration in association with a positive culture for *B*. *pseudomallei*. This was further defined as recrudescence if it occurred after completion of the intensive phase of intravenous therapy, during the time that oral eradication therapy was recommended, whether this therapy was being taken or not. In the absence of molecular typing, disease recurrence was defined as relapse–rather than reinfection—if it occurred within two years of the recommended completion date of oral therapy, whether this therapy had been taken or not [13]. Australian Bureau of Statistics population data were used to determine disease incidence [11]; while Bureau of Meteorology data were used to explore the link with rainfall [14].

### Statistical analysis

Data were de-identified, entered into an electronic database and analysed with statistical software (Stata 10). Groups were analysed using the Kruskal-Wallis and chi-squared tests.

### Ethics review

The study received ethical approval from the Far North Queensland Human Research Ethics Committee (HREC/15/QCH/46–977), as per this approval this retrospective study with anonymized patient data did not obtain individual patient consent.

## Results

**Fig 3:**
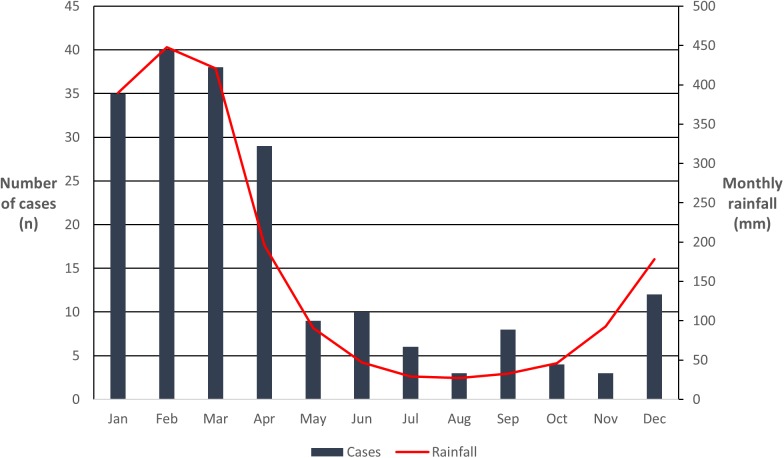
Relationship between the monthly rainfall and the timing of clinical presentation.

**Fig 4:**
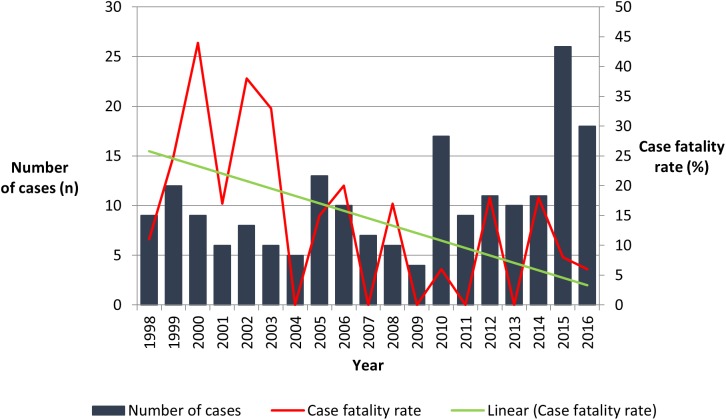
Year of presentation and case-fatality rate.

Over the course of the study period the annual incidence was higher in the Torres Strait (33.1/100,000 population) and on Cape York (12.1/100,000 population) than in Cairns and surrounds (1.7/100,000 population) (Fig 1). Although the number of cases varied from four to 26 per year, in every year they clustered around the region’s December to April wet season (Figs 3 & 4).

During the study period, 197 individuals (median age 50 years; range 2 days to 92 years) had *B*. *pseudomallei* isolated (Fig 4); 147 (75%) were male and 50 (25%) were female; 117 (59%) identified as ATSI, 80 (41%) did not identify as ATSI. The mean annual incidence in the ATSI population over the study period was 14.0/100,000 population versus 1.9/100,000 population in the non-ATSI population. ATSI patients were younger (median 46 years (interquartile range (IQR): 38–56) than non-ATSI patients (median (IQR): 59, 43–69 years) (p < 0.001).

Of the 162 patients with complete data, only 14 (8.6%) had no apparent risk factor for the disease. In patients with sufficient data to determine if the risk factor was present, 102/182 (56%) had diabetes mellitus, 86/166 (52%) had hazardous alcohol use and 28/180 (16%) had chronic kidney disease. Chronic lung disease was present in 26/169 (15%), while immunosuppression or malignancy was present in 27/168 (16%) (Table 1). Of the 162 patients with complete data, 85 (52%) had more than one risk factor. The ATSI population were more likely to have diabetes and chronic kidney disease, while lung disease was more common in the non-ATSI population. The ATSI population were more likely to have multiple risk factors for the disease while the non-ATSI population was more likely to have no documented risk factor (Table 2).

**Table 1:** Disease risk factors and their relationship with mortality.

Risk Factor		Number N	Deaths N (%)	Survived N (%)	P ^a^
Male	Yes	147 (75)	19 (13)	128 (87)	0.59
	No	50 (25)	8 (16)	42 (84)	
ATSI	Yes	117 (59)	22 (19)	95 (81)	0.01
	No	80 (41)	5 (6)	75 (94)	
Diabetes mellitus	Yes	103 (56)	12 (12)	90 (88)	0.71
	No	80 (44)	8 (10)	72 (90)	
Hazardous alcohol intake	Yes	86 (51)	9 (10)	77 (90)	0.92
	No	80 (49)	8 (10)	72 (90)	
Chronic kidney disease	Yes	28 (16)	7 (25)	21 (75)	0.004
	No	152 (84)	11 (7)	141 (93)	
Chronic lung disease	Yes	26 (16)	3 (11)	23 (89)	0.60
	No	143 (84)	12 (8)	131 (92)	
Immunosuppression	Yes	17 (10)	2 (12)	15 (88)	0.59
	No	151 (90)	12(8)	139 (92)	
Malignancy	Yes	14 (8)	1 (7)	13 (93)	0.87
	No	154 (92)	13 (8)	141 (92)	
No risk factor	Yes	14 (8)	2 (14)	12 (86)	0.43
	No	148 (92)	12 (8)	136 (92)	
Overall		197	27 (14)	170 (86)	-

^a^ For the association with death calculated using chi square.

ATSI: identifying as Aboriginal or Torres Strait Islanders.

**Table 2:** The presence of risk factors for disease, stratified by ATSI status.

	Number ^a^	Remote location ^b^	Diabetes mellitus	Hazardous alcohol consumption	Chronic kidney disease	Chronic lung disease	Multiple risk factors	No recognised risk factor
**ATSI**	117	92/117 (79)	77/105 (73)	52/93 (56)	21/103 (20)	7/95 (7)	54/90 (60)	3/90 (3)
**Non-ATSI**	80	22/80 (28)	25/77 (32)	34/73 (47)	7/77 (9)	19/74 (26)	31/72 (43)	11/72 (15)
**P value** ^**c**^		<0.001	<0.001	0.23	0.04	0.001	0.03	0.007

^a^ Incomplete data for some risk of the factors.

^b^ Outside of metropolitan centres.

^c^ Determined using Chi-square.

ATSI: identifying as Aboriginal or Torres Strait Islander.

Of the 197 patients, 145 (74%) were bacteraemic. Septic shock was present on admission or early in the hospitalisation in 58 (34%) of the 175 patients in whom this could be determined (Table 3). Pneumonia was the most common presentation, occurring in 101 (61%) of the 166 patients with chest imaging (Table 4). An abscess was present in 89 (53%) of the 166 patients who had either complete imaging or an operation report documenting a culture positive aspirate; 15 (9%) had abscesses in multiple organs.

**Table 3:** Disease severity, outcome and supportive care over the course of the study.

Variable	1998–2002	2003–2006	2007–2011	2012–2016	Total
	Number (%)	Number (%)	Number (%)	Number (%)	Number (%)
Died	12/44 (27)	6/34 (18)	2/43 (5)	7/76 (9)	27/197 (14)
Bacteraemic	30/44 (68)	23/34 (68)	34/43 (79)	58/76 (76)	145/197 (74)
ICU admission	11/29 (38)	14/31 (45)	12/39 (31)	21/76 (28)	58/175 (33)
Septic shock	11/29 (38)	15/31 (48)	12/39 (31)	20/76 (26)	58/175 (33)
Mechanical ventilation	10/28 (36)	14/31 (45)	11/39 (28)	12/76 (16)	47/174 (27)
RRT	3/24 (13)	5/30 (17)	5/39 (13)	4/76 (5)	17/169 (10)

ICU: Intensive Care Unit; RRT: Renal replacement therapy.

**Table 4:** Clinical manifestations and organ involvement.

Clinical manifestation	Yes (%)	No (%)
Bacteraemia	145 (74)	52 (26)
Abscess in any location ^a^	89 (53)	77 (47)
Pneumonia ^b^	101 (61)	65 (39)
Skin and soft tissue involvement ^c^	28 (17)	136 (83)
Liver or spleen involvement ^c^	28 (18)	127 (82)
Genitourinary involvement ^c^	38 (24)	121 (76)
Musculoskeletal involvement ^c^	26 (16)	133 (84)
Central nervous system involvement ^c^	6 (4)	148 (96)

^a^ Includes lung abscesses and empyema.

^b^ Determined by review of chest X-ray.

^c^ Determined on review of medical records, microbiological specimens and medical imaging. Note: patients could meet criteria for inclusion in multiple categories.

**Fig 5:**
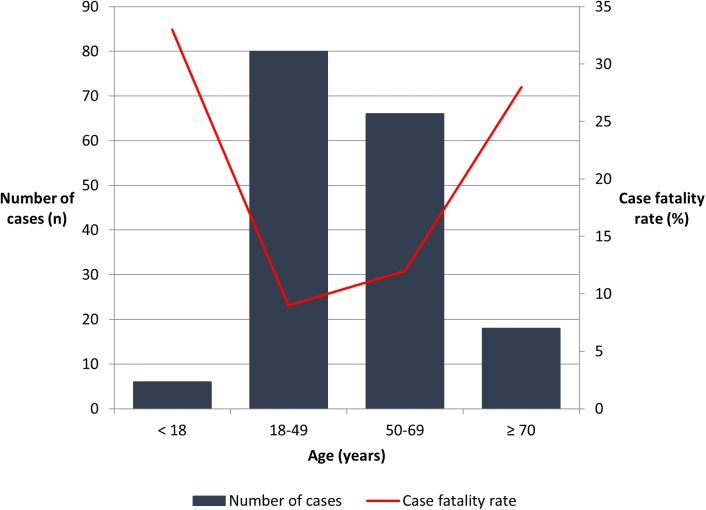
Age at presentation and case-fatality rate.

The disease was directly responsible for 27 (15%) deaths during the study period although the case fatality rate declined over time (test for trend p = 0.009): in the first 5 years of the study period 12/44 (27%) died, compared with 7/76 (9%) in the last 5 years. There was no significant increase in the rates of ICU admission and the use of vasopressors, RRT or mechanical ventilation over the study period to explain this improvement (Table 3). Nine (33%) of the 27 deaths, occurred within the first 48 hours of the patient’s presentation and 13 (48%) occurred within the first seven days. There were 22 (19%) deaths among the 117 ATSI patients versus five (6.3%) in the 80 non-ATSI patients (p = 0.01). While there was a trend towards the ATSI population requiring more critical care support including admission to ICU (38% versus 27% in non-ATSI patients, p = 0.11), mechanical ventilation (32% versus 21% in non-ATSI patients, p = 0.11) and RRT (13% versus 7%, p = 0.18) this did not achieve statistical significance. All three patients under ten years of age died. In adults, the case fatality rate increased with increasing age (p = 0.03) (Fig 5). The only comorbidity associated with death in univariate analysis was chronic kidney disease (Table 1).

In the 155 patients with complete data who survived the intensive intravenous therapy phase, there were eleven (7.1%) with a microbiologically confirmed recrudescence or relapse (Table 5). Of the 155 patients, 107 (69%) received intravenous therapy for a period that was greater than or equal to that recommended in the Darwin guidelines [10]. Nine (8.4%) of these 107 patients had either recrudescence (seven) or relapse (two); two of these patients died, although in one of these cases, outpatient parenteral therapy was interrupted for 72 hours during a category 5 cyclone. Two (4.2%) of the 48 patients with an intensive treatment duration of less than that recommended in the Darwin guidelines had recurrence. There was one recrudescence and one relapse; both patients survived.

**Table 5:** Cases of disease recurrence with potential explanations.

Age/Sex	Adherent to Darwin Recommended duration of intravenous therapy [10] (Number of weeks received)	Recrudescence or relapse	Primary site of disease	Abscess site	Adequate source control	Documented adherence to oral eradication therapy	Died
18M	No (0.5)	Recrudescence	Parotitis	Parotid	Yes	No	No
29M	No (1)	Relapse	Skin	Skin/soft tissue	Yes	No	No
31M	Yes (4)	Recrudescence	Disseminated	Skin/liver/pancreas	No ^a^	No ^f^	No
43M	Yes (4)	Recrudescence	Bacteraemia	Prostate	No ^b^	No ^f^	No
61M	Yes (6)	Recrudescence	Pneumonia	Prostate	No ^c^	Yes ^f^	No
38M	Yes (6)	Recrudescence	Pneumonia & bacteraemia	Liver	No ^d^	No	No
50F	Yes (4)	Recrudescence	Pneumonia	Nil	Not required	Unknown ^e^^,^ ^f^	Yes
26M	Yes (2)	Recrudescence	Pneumonia	Nil	Not required	No	No
31F	Yes (7)	Relapse	Disseminated	Septic arthritis	Yes	No	No
47F	Yes (6)	Relapse	Pneumonia	Nil	Not required	No	Yes
64F	Yes (2)	Recrudescence	Skin	Skin/soft tissue	No ^g^	No	No

^a^ Managed conservatively.

^b^ Patient declined surgical intervention.

^c^ Initial imaging revealed unilateral swelling but unamenable to drainage.

^d^ Unamenable to radiologically guided drainage.

^e^ Missed 72 hours of intravenous therapy due to cyclone (although course extended).

^f^ Recrudesced within one month of ceasing intensive phase therapy.

^g^ Surgery deferred in favour of antibiotics.

In the eleven patients with a microbiologically confirmed recrudescence or relapse, this could be explained by suboptimal source control in four patients and poor adherence to prescribed eradication therapy in another five. Recrudescence also occurred in one patient with an ulcerated skin lesion in whom surgical debridement was considered, but not performed; he also subsequently had poor adherence to eradication therapy (Table 5).

There were a further two patients who had a recurrence of melioidosis several years after their initial eradication therapy. Both patients had an intensive phase of treatment less than that recommended in the Darwin guidelines and neither was adherent to eradication therapy. In the absence of molecular typing, it was not possible to determine whether these two cases represented relapse or reinfection, although reinfection is suspected based on the interval durations of relapse and reinfection seen in other studies [8, 13]. One–with a recurrence four years after his initial presentation–had poorly controlled diabetes, ongoing hazardous alcohol use, end stage renal disease requiring haemodialysis and hepatocellular carcinoma; the other—with disease recurrence five years after his initial presentation—had poorly controlled diabetes (glycosylated haemoglobin persistently above 10%).

## Discussion

Melioidosis in Far North Queensland is, very largely, a seasonal, opportunistic disease of patients with specific co-morbidities. Patients are commonly bacteraemic and frequently require ICU admission. Even with ICU support, the disease has a high attributable case-fatality rate, although this is declining. ATSI patients bear the greatest burden of disease, develop the disease at a younger age and have a higher case fatality rate, despite similar access to ICU support. The disease has a very similar epidemiology and clinical presentation to that in the Northern Territory, but unlike the Territory patients, those in Queensland receiving extended courses of intensive, intravenous antibiotic therapy still had relatively high rates of disease recurrence. These recurrences were often explained by suboptimal source control and poor adherence to the eradication phase of treatment.

Almost 80% of clinical presentations occurred during the region’s wet season, a very similar figure to that seen the hyper-endemic Top End of the Northern Territory [4, 15] and northeast Thailand [16]. This association with rainfall reflects the increased *B*. *pseudomallei* load in the top soil and surface water which is thought to result from the rising water table [17], improved bacterial survival in soil with a high moisture content [18, 19], and more vegetation for colonisation [19]. However, while presentations are linked strongly to rainfall, the importance of other patient and environmental factors is emphasised by the fact there were only three cases seen in patients from the Cassowary Coast Region during the entire study period. This is notable because the region has the highest mean rainfall in Australia, a population of over 30,000 and Australia’s highest incidence of leptospirosis, a disease that is also acquired percutaneously [1, 20–22].

Two of the largest cyclones ever recorded in the region occurred during the study period (Cyclone Larry (March 2006) and Cyclone Yasi (February 2011)). While adverse weather events have been linked to an increase in melioidosis presentations [23], in the three months following Cyclone Larry there were only four cases and after Cyclone Yasi only three; two of these seven cases occurred in the Torres Strait, over 800km to the North. The eye of both cyclones passed through the Cassowary Coast region, but on neither occasion was there a case locally.

The incidence rates reported in the Torres Strait are amongst the highest ever described in Australia [24]. This presumably reflects a combination of an increased prevalence of the comorbidities linked to melioidosis in Torres Strait residents—83% of whom identify as ATSI–and increased recreational and occupational exposure. Whether there are other local factors in the Torres Strait which are contributing to this relatively high incidence (soil characteristics, local precipitation and land surface temperature variability and vegetation differences) is a subject of ongoing study.

Almost all the patients in the series had a recognised risk factor for the disease, including diabetes mellitus, hazardous alcohol use, chronic kidney disease and chronic lung disease, reinforcing the notion that melioidosis is predominantly an opportunistic infection in an at risk population [4, 6, 25, 26]. Indeed, only a few patients had no risk factor for the disease; of the 14 patients without any documented risk factors, four were children and three had clear inoculation events. Our data concords with an earlier Australian study that has shown male sex to be an independent risk factor for melioidosis, presumably due to increased exposure risk [27]. The rate of bacteraemia (74%) seen in this study is higher than that seen in the prospective survey from the Northern Territory (55%) [28]. This may be related to our study population having a higher rate of risk factors for bacteraemia (ATSI ethnicity, older age, diabetes, alcohol misuse and kidney disease) than the Northern Territory cohort.

Unfortunately, 23 patients had incomplete risk factor data available; this was usually the result of destroyed charts. Among these 23 patients, there were 10 deaths, which represented over one third of the deaths in the cohort. This missing data limited our ability to perform meaningful statistical analysis of the association between individual comorbidities and mortality. No comorbidity, other than chronic kidney disease, was associated with increased risk of death.

Across the entire region, the incidence of melioidosis was over seven times higher in the ATSI population than in the non-ATSI population. This is almost certainly at least partly explained by the increased prevalence of the comorbidities associated with melioidosis in the ATSI population. In Far North Queensland, the prevalence of diabetes mellitus is three-fold higher, alcohol abuse two-fold higher and chronic kidney disease six-fold higher in the ATSI population than the non-ATSI population [29–31]. It has also been suggested that the higher rates of melioidosis in the ATSI population may relate to increased exposure [4]. Not only did the ATSI population bear a greater burden of disease, they also had disease at a younger age and a higher case-fatality rate. This reflects the stubbornly persistent “gap” in health outcomes between ATSI and non-ATSI populations in Australia [32], strongly linked to the socioeconomic determinants of disease and reduced access to health care. In this series, the ATSI patients were more likely to have multiple risk factors for the disease that not only predisposed them to developing melioidosis but also—through decreasing physiological reserve—presumably contributed to their worse outcomes.

The low incidence in children echoes findings from the Northern Territory [33]. These Australian data are notable, as the disease is seen more frequently in children in some parts of South East Asia [34, 35], which may reflect the fact that unchlorinated domestic water supplies are contaminated with *B*. *pseudomallei* in these locations [6, 33, 36]. The reduced likelihood of exposure to *B*. *pseudomallei* in drinking water is also reflected in the finding that only two patients had parotid abscesses. Although the literature suggests a low case-fatality rate in children [33, 34], all three children in the series under the age of ten (ages 0, 5 and 6 years old), died from the disease within 48 hours of presentation. None of the children had identifiable risk factors, although one child died within the neonatal period. Elderly patients also had a higher mortality, although this presumably reflects the decreased physiological reserve of older individuals.

Recognising that adherence to the extended oral eradication phase of treatment is frequently suboptimal, Darwin investigators have recently proposed a new treatment approach. This is based on the observation that progressive lengthening of duration of intensive intravenous therapy at their institution has been associated with a reduction in relapse rates, even though these patients still have a high rate of non-adherence to the eradication phase [10]. If these findings were reproducible, this would influence International treatment guidelines and support a longer course of intensive intravenous therapy, which might potentially allow the oral eradication phase of therapy to be abbreviated or avoided altogether. Our study provided an opportunity to assess the Darwin approach but we were unable to demonstrate that a longer duration of intensive intravenous therapy in our patients reduced disease recurrence. Disease recurrence was no lower in the patients who had completed a period of intensive intravenous therapy consistent with the Darwin guidelines. Indeed, the only deaths in patients with recurrent disease occurred in patients with prolonged intravenous therapy (although this is almost certainly the result of unfavourable clinical characteristics—disease burden and patient comorbidities—rather than a complication of the prolonged therapy). Instead, disease recurrence was explained more commonly by inadequate source control and an inadequate duration of oral eradication therapy secondary to non-adherence. The first point is not surprising as source control is fundamental to the management of infection [37] while an inadequate duration of oral therapy is consistent with findings from a Thai series where duration of oral therapy was identified as one of the strongest predictors of relapse [9]. Patients who are not able to complete an adequate duration of prescribed oral eradication therapy might also be anticipated to be a population less inclined to engage with medical services. This would preclude optimal management of the comorbidities that predisposed them to the disease, potentially facilitating perpetuation and reactivation of this opportunistic pathogen. In the absence of molecular typing, our inability to differentiate early reinfection from relapse will influence our treatment outcome comparisons. However, reinfection is relatively uncommon within the 2-year window of our case definition for relapse [13]. While our findings in a retrospective cohort do not preclude the possibility that an extended period of intensive intravenous therapy will have salutary effects on patient outcomes, they underline the fact that frequently the more prosaic, but fundamental, aspects of disease management should not be overlooked. A prospective, randomised controlled trial may help better define the optimum durations of intravenous therapy for melioidosis.

The case-fatality rate fell impressively during the study period, echoing findings from Darwin where a similar improvement was attributed predominantly to improvements in the ICU management of the sepsis [38]. Indeed, this is a trend that has been seen Australia-wide in all bacterial sepsis, not just in melioidosis [39]. However, unlike Darwin, granulocyte colony-stimulating factor is not routinely used in the management of patients critically ill with melioidosis in Far North Queensland [38]. The fact that a third of all deaths occurred within the first 48 hours of presentation emphasises the importance of prompt ICU care. Interestingly, the overall case fatality rate was no higher in the patients coming from remote locations than those patients living within 30 minutes of Cairns hospital. This likely reflects the ability of clinicians working in remote parts of Far North Queensland to recognise critically ill patients, and the efficiency of retrieval services in transporting these patients long distances to receive tertiary level support. Other factors that may have contributed to the improvement in mortality over the period of the study include improvements in imaging which may have identified previously unrecognised foci. Improved access to digital education resources over the study period also permitted the promulgation of guidelines for the recognition and management of the disease, which may also have contributed to superior outcomes.

The relatively high disease burden in remote Cape York and the Torres Strait communities may help guide empiric antibiotic therapy in patients presenting with sepsis in these communities. Clearly, meropenem should be in the essential medicines list for the Far North Queensland retrieval service given the recognition that delays in the administration of appropriate antibiotic therapy in septic patients is linked to increased mortality [40]. In the appropriate clinical situation empiric therapy that covers the possibility of melioidosis may be lifesaving, however, it should be noted that even in endemic areas such as Far North Queensland, other pathogens are a more common cause of community-acquired sepsis. For example, over the last five years of the study period, there were 76 cases of *B*. *pseudomallei* bacteraemia, but during the same time period Cairns Hospital managed 661 cases of community-acquired *Escherichia coli* bacteraemia and 389 cases of community-acquired *Staphylococcus aureus* bacteraemia.

Our study has limitations. Its retrospective nature resulted in incomplete data collection, limiting multivariate statistical analysis in particular. Comparisons with results from Darwin are particularly fraught given the prospective nature of data collection in that centre, although the independent dataset permits external validation–at least to some degree–of the recommendations of that high-volume unit.

In conclusion melioidosis in Far North Queensland is an opportunistic infection of patients with specific comorbidities that presents predominantly in the wet season. Differences in incidence across the region almost certainly reflect to some degree differences in exposure and risk factor prevalence, however as yet undefined environmental factors appear to make a significant contribution. The ATSI population who have a higher prevalence of the risk factors for melioidosis, bear the greatest burden of the disease. Disease recurrence is higher in patients with inadequate source control and suboptimal adherence, factors which are not necessarily mitigated by extending the duration of intravenous therapy. Survival is improving as is the case with other aetiologies of bacterial sepsis, however as the majority of deaths occur early in the patient’s hospitalisation prompt consideration of the diagnosis and administration of appropriate antibiotic therapy is critical to reduce case-fatality rates further. Prospective multi-centre studies will help determine optimal disease management strategies.
